# Local language proficiency of fourth-year medical students at the University of the Free State

**DOI:** 10.4102/safp.v65i1.5800

**Published:** 2023-12-21

**Authors:** Peter Ngobeni, Maleho Sebolai, Licham Hlotshana, Tshwanelo Henani, Siphosomusa Masango, Smangaliso Hlongwane, Samkelo Ngqulu, Thabelo Makhaba, Carl van Ramesdonk, Gina Joubert

**Affiliations:** 1 Department of Family Medicine, Faculty of Health Sciences, University of the Free State, Bloemfontein, South Africa; 2 Department of Biostatistics, Faculty of Health Sciences, University of the Free State, Bloemfontein, South Africa

**Keywords:** communication, language, proficiency, medicine, student, patient, health care

## Abstract

**Background:**

Language proficiency is beneficial for doctor–patient communication and health outcomes. Poor communication can lead to misdiagnosis by the doctor and/or non-adherence from the patient. This study aimed to evaluate medical students’ proficiency in the most commonly spoken local languages.

**Methods:**

This cross-sectional study was conducted in the class of 119 fourth-year medical students at the University of the Free State (UFS) in 2019. Students’ proficiency was tested for Sesotho and Afrikaans, as these are the most widely spoken languages in the Free State province. The study consisted of two phases: completing a self-administered questionnaire where students self-rated their proficiency in the two languages, followed by telephonic interviews consisting of a series of proficiency-testing questions.

**Results:**

Of the 119 fourth-year medical students at UFS, 96 (80.7%) completed the self-administered questionnaires. Forty-six students (47.9%) rated themselves as either advanced or proficient in Afrikaans, whereas only 23 students (23.9%) rated themselves as advanced or proficient in Sesotho. Only 28 students were subsequently interviewed. Their actual language proficiency matched their self-rating.

**Conclusion:**

The findings suggest a need for language skills training improvement in the curriculum for undergraduate medical students for languages most commonly encountered locally. We also found that students report their language capabilities accurately.

**Contribution:**

The research findings reinforce the need for language skills training in the curriculum of undergraduate medical students regarding languages commonly encountered in the local area.

## Introduction

There is a strong correlation between the language proficiency of doctors and the quality of health care patients receive.^1^ The National Health Act 61 of 2003 states that health providers ought to communicate health-related matters with their patients in a language that the patients understand and in a manner that considers the patients’ level of literacy.^2^

Misunderstanding between doctor and patient because of limited language concordance leads to misdiagnosis of diseases and decreases the likelihood of patients to adhere to treatment regimens; this also decreases the patient’s trust in the doctor, eventually leading to an ineffective treatment regimen.^3,4,5^ Some patients have reported receiving minimal attention because of their inability to communicate with their doctors fluently, compromising their safety, comfort and satisfaction.^3,4,6,7^ A study from Boston, United States (US), found that patients with low English proficiency were more likely to have poorly controlled hypertension than those with adequate English proficiency.^8^ Levin emphasised in his literature review that language difficulty resulted in a decreased understanding of diagnoses, medication and follow-up, as well as decreased adherence to medical advice.^9^ His research found that most patients believed that language and cultural barriers were the most significant impediment to healthcare.^9^

Patients’ ability to comprehend the health language, their adherence to curative care and satisfaction have been shown to improve with language concordance.^3^ Language concordance reduced language-related misconceptions.^10^

A study from California, US, found that the increased effectiveness of the treatment of patients with diabetes resulted from an improved patient-practitioner relationship aided by the physicians’ high proficiency in the patients’ language.^10^ Studies in the US regarding Spanish-speaking patients reported enhanced cooperation and an increased likelihood of patients sharing relevant diagnostic information when treated in their own language.^11,12^

Poor local language proficiency also has consequences for doctors. It can affect their confidence, competence and work satisfaction. The study from California found that linguistic impediments reduce health workers’ work effectiveness and ability to make holistic care available, as it consumes more time to make a precise medical diagnosis, which leads to frustration.^10^ Another US study found misdiagnosis as a result of language discordance.^13^ A study from the United Kingdom reported that in a clinical setting, healthcare professionals felt that the most problems arose from them not understanding idioms and colloquial language used by patients. Such misunderstandings could have adverse effects on patient safety.^14^

Doctors in the Free State province, South Africa, lacked adequate language proficiency in Sesotho, the most common local language, according to a 2006 study,^15^ despite most respondents being trained in the province. The study recommended better language training during the undergraduate medical programme.^15^

Language proficiency is not only a problem after graduation. Clinical training for medical students can be challenging if they lack language proficiency. A study from Qatar^16^ showed that healthcare students who were less diverse in language skills faced more frustration and stress in the clinical setting. Abdelrahim et al.^16^ also reported that language discordance could lead to lower quality of health information, higher cost implications and increased health risks. Therefore, providing comprehensive language teaching for health students in clinical training is essential. A study^17^ from KwaZulu-Natal, South Africa, found that teaching students introductory isiZulu in their first year enhanced their training experience. Even though they could not communicate fluently in isiZulu by their third year, they were still positive and wanted more language training.

Medical schools have the responsibility to adapt their curriculum to prepare students for the language challenges they will face in their clinical years and beyond. However, before making any changes, the proficiency levels of students need to be assessed.

### Aim and objectives

This study aimed to evaluate the proficiency of fourth-year medical students at the University of the Free State (UFS) in two local languages, Sesotho and Afrikaans.

The objectives included determining the following:

- students’ home language, languages studied at school and languages acquired elsewhere
- students’ self-perceived language proficiency in Sesotho and Afrikaans
- students’ language proficiency in Sesotho and Afrikaans.

## Methodology

### Study design, population and sampling strategy

This was a cross-sectional study. The sample included all 119 fourth-year medical students registered at the UFS in the second semester of 2019. The fourth year of the training programme is the first full year of clinical training.

### Setting

The UFS is situated in central South Africa. The School of Clinical Medicine educates from the Main Campus in Bloemfontein but collaborates with satellite campuses throughout the Free State. The language of instruction at the UFS is English. During their first semester, medical students receive introductory language training in Sesotho and Afrikaans regarding medical terminology and phrasing. During a 2-h session, the lecturer instructs students on how to set up probes between two or more people conversing in a language the student does not understand. After that, the student has a self-directed learning component of about 2 weeks, where they get to practice a greeting ritual in preparation for their clinical visit. According to the Central Statistical Services’ 2011 South African census, Sesotho (71.9%) and Afrikaans (10.9%) are the most widely spoken home languages in the Free State.^18^

### Measurement

The study included two phases: completing a self-administered questionnaire in a classroom setting and thereafter a telephonic interview to assess language proficiency. During the first phase, all students in the fourth-year class were requested to complete a self-administered hard-copy questionnaire. The purpose of this tool was to determine the participants’ home language(s), additional languages spoken, first and additional languages taken at primary and secondary level and their self-rated level of proficiency in the most commonly spoken languages in the province, Sesotho and Afrikaans. ‘First language’ refers to the language medium in which learning and teaching, including assessment, takes place as stipulated by the Department of Basic Education.^19^ ‘Additional languages’ are the languages done at high school at the First Additional level as stipulated by the Department of Basic Education.^19^

The second phase was a telephonic interview to assess the participants’ actual proficiency. The questions for the interview were four sets of equivalent questions in Sesotho and Afrikaans. The English formulation of these questions was:

- How would you tell a patient to describe the pain they are feeling?
- How would you describe to a patient how to take medication [therapeutic effect, side effect and contra-indications]?
- What specialty do you think of taking up once you qualify and why?
- What specialty is your least favourite and why?

In Sesotho:

- *O ka reng ho mokudi hao batla ao hlalosetse bohloko boo a tletlebang ka bona?*
- *O ka reng hao hlalosetsa mokudi ka mekgwa eo a tlamehang ho e latela ha nka dipidisi?*
- *Hao o se o ena le lengolo la hao la bongaka, o ka rata hoba ngaka ya eng mme hobaneng ore jwalo? [mohlala ya masapo,boko kapa ya pelo]*
- *Ke mofuta ofeng wa bongaka boo o sa borateng hakaalo, hobaneng o ikutlwa jwalo?*

In Afrikaans:

- *Hoe sou jy ‘n pasiënt vertel om die pyn wat hy/sy voel te beskryf?*
- *Hoe sou jy vir ‘n pasiënt beskryf hoe om hul medikasie te neem? [terapeutiese effek, newe-effekte en kontra-indikasies]*
- *Watter spesialiteit dink jy aan om op te neem wanneer jy kwalifiseer en hoekom?*
- *Watter spesialiteit is jou minste gunsteling en hoekom?*

Students’ responses were graded by the student researchers who are fluent in these languages, using a four-level scale: 1 = participant cannot understand the question / no response; 2 = participant completely misunderstood the question; 3 = participant understands the question but struggles to express; 4 = participant understands question and answers appropriately.

### Pilot study

The researchers completed a pilot study on 10 first-year medical students, of whom eight agreed to participate in the study’s second phase. Issues identified in the questionnaire were corrected. The first-year students were asked to complete the edited questionnaire again, and no other errors were identified.

### Data analysis

All coded data were captured on an Excel spreadsheet. Data were analysed by the Department of Biostatistics, Faculty of Health Sciences, UFS, using SAS Version 9.4. Results were summarised by frequencies and percentages (categorical variables) and medians and interquartile ranges (IQR) (numerical variables because of skew distributions). Spearman rank correlations were calculated between self-rated proficiency and actual proficiency score.

### Ethical considerations

The protocol was approved by the Health Sciences Research Ethics Committee of the UFS (UFS-HSD2019/0525/0110). Gatekeeper approval from the Dean of the Faculty of Health Sciences, the Head of the School of Clinical Medicine, the Dean of Student Affairs and the Vice-Rector of Research was obtained. Participants’ identities remained confidential. Before participation, they gave written informed consent and were informed that participation was voluntary.

## Results

Of the 119 fourth-year medical students at the UFS, 96 completed the self-administered questionnaires (response rate 80.7%). Of the 96, 41 (42.7%) consented to the telephonic interviews. Of these 41 students, 28 (68.3%) answered when contacted to participate in the telephonic interviews. As shown in Table 1, there were more females in the class than males, and females were the majority of the participants in the two phases. The median age of the participants was 23 years (range 21–34 years).

**TABLE 1:** Gender distribution of the fourth-year class and the participants in Phases I and II of the study.

Gender	Number in class (*n* = 119)		Phase I (*n* = 96)		Phase II (*n* = 28)	
	*n*	%	*n*	%	*n*	%
Male	52	43.7	43	44.8	10	35.7
Female	67	56.3	53	55.2	18	64.3

In Table 2, the predominant home language (the primary language spoken at home) among the participants was Afrikaans (33.3%), followed by English (25.0%) and Sesotho (20.8%). English was the predominant first language among the study participants (62.5%), followed by Afrikaans (32.3%) and Sesotho (2.1%). The predominant first additional language among the study participants was Afrikaans, followed by English and Sesotho. The ‘other languages’ are defined as languages not learned as part of the school curriculum or spoken at home as the primary language.

**TABLE 2:** Language profile of participants in Phase I (*n* = 96).

Variable	English		Sesotho		Afrikaans		Other official South African languages	
	*n*	%	*n*	%	*n*	%	*n*	%
Home languages†	24	25.0	20	20.8	32	33.3	25	26.0‡
First language in school	60	62.5	2	2.1	31	32.3	9	9.3§
First additional language in school (*n* = 83)	34	41.0	4	4.8	43	51.2	9	9.3¶
Other languages able to converse in	8	8.3	2	2.1	2	2.1	19	19.8††

† , Primary language(s) spoken at home.

‡ , IsiZulu (eight students), isiXhosa and Setswana (seven students each) were the most common other South African languages.

§ , IsiXhosa (four students) the most common.

¶ , IsiXhosa (five students) the most common.

†† , IsiZulu (11 students) the most common.

Of the 37 participants who indicated how they learned their first additional language, family was the most common source (32.4%), followed by school (29.7%), ‘picked it up’ (21.6%) and university (18.9%). The remaining participants learned from friends and television.

The largest percentage of the study participants in Phase I rated themselves as beginners or elementary (54.2%) in Sesotho, whereas the largest percentage rated themselves as either advanced or proficient (47.9%) in Afrikaans (Table 3). Participants in Phase II were more likely to rate themselves as proficient in Sesotho and less likely to rate themselves as proficient in Afrikaans than those participating in Phase I.

**TABLE 3:** The self-rated proficiency of the participants in Sesotho and Afrikaans.

Language proficiency level	Sesotho				Afrikaans			
	Phase I (*n* = 96)		Phase II (*n* = 28)		Phase I (*n* = 96)		Phase II (*n* = 28)	
	*n*	%	*n*	%	*n*	%	*n*	%
None	14	14.6	1	3.6	17	17.7	9	32.1
Beginner/elementary	52	54.2	14	50.0	21	21.9	6	21.4
Intermediate	7	7.3	4	14.3	12	12.5	4	14.3
Advanced/proficient	23	23.9	9	32.1	46	47.9	9	32.1

Note: None, no proficiency; Beginner, use simple phrases for basic needs; Elementary, use the language for everyday activities; Intermediate, have simple conversations about familiar topics; Advanced, express yourself fluently in any situation; Proficient, speak with complete mastery.

Table 4 indicates the scores for the individual questions of the interview assessment. The median overall scores on the scale from 4 to 16 were 9 (Sesotho) and 10.5 (Afrikaans) with IQR 4 to 16 for both languages. Eight students (28.6%) were completely proficient (scored 16/16) in Sesotho and eight (28.6%) in Afrikaans, whereas 10 (35.7%) had no knowledge of the language (scored 4/16) in Sesotho and 10 (35.7%) in Afrikaans. One participant scored 4 in both languages, and the most proficient student scored 15 and 16 in the two languages, respectively.

**TABLE 4:** The scores of the participants from the Sesotho and Afrikaans telephonic interviews (*n* = 28).

Score code	Question 1				Question 2				Question 3				Question 4			
	Sesotho		Afrikaans		Sesotho		Afrikaans		Sesotho		Afrikaans		Sesotho		Afrikaans	
	*n*	%	*n*	%	*n*	%	*n*	%	*n*	%	*n*	%	*n*	%	*n*	%
1	12	42.9	12	42.9	12	42.9	12	42.9	15	53.6	11	39.3	15	53.6	14	50.0
2	1	3.6	1	3.6	0	0	2	7.1	0	0	1	3.6	0	0	0	0
3	4	14.3	3	10.7	5	17.9	3	10.7	3	10.7	6	21.4	4	14.3	6	21.4
4	11	39.3	12	42.9	11	39.3	11	39.3	10	35.7	10	35.7	9	32.1	8	28.6
Median answer†	3	-	3	-	3	-	2.5	-	1	-	3	-	1	-	2	-

† , 1, participant cannot understand the question/no response; 2, participant completely misunderstood the question; 3, participant understands the question but struggles to express; 4, participant understands question and answers appropriately.

Table 5 summarises the actual language proficiency tested in the interview, grouped according to the students’ self-rated language proficiency. Students with higher self-rated language proficiency had higher actual language proficiency scores. The Spearman rank correlation coefficients between self-rated language proficiency and actual language proficiency score were 0.93 for Sesotho and 0.96 for Afrikaans.

**TABLE 5:** The scores from the Sesotho and Afrikaans telephonic interviews according to various self-rated proficiency levels.

Self-rated proficiency level†	Description	Sesotho (*n* = 28)			Afrikaans (*n* = 28)		
		Number of participants	Score on interview‡		Number of participants	Score on interview‡	
			Median	IQR		Median	IQR
0	None	1	4	4–4	9	4	4–4
1–2	Beginner/elementary	14	4	4–6	6	5	4–10
3	Intermediate	4	12	10.5–14.5	4	12	11.5–13
4–5	Advanced/proficient	9	16	16–16	9	16	16–16

IQR, interquartile range.

† , The original six levels were grouped into four categories for ease of comparison.

‡ , Possible score range 4 to 16 (4 questions each scored 1–4).

## Discussion

The high response rate in Phase I and the gender distribution, which reflects the study population, increase the representativeness of the participants from Phase I. The participants had diverse home languages, including Sesotho, Afrikaans, English and other official languages. However, the majority received their school education in English. Most of those with an additional language learned from their family, school or ‘picked it up’ in the community. Only a small percentage (18.9%) learned an additional language informally while at university.

Although Phase II of the study had a much lower response rate, the study confirmed that students have insight into their own language capabilities. This insight on the part of the students should enhance attempts to improve students’ language proficiency. In a study of final-year medical students at the University of KwaZulu-Natal, South Africa, students were predominantly positive regarding the need for doctors to be at least bilingual.^20^ A different study by the same research team found that students in their third year had positive attitudes regarding communication competence 2 years after the introductory training of a year-long module in isiZulu, even though they did not yet feel competent in having conversations in the additional language.^17^ Students are aware of their shortcomings and generally are open to additional training.

The language proficiency of the majority of UFS medical students in the most widely spoken languages in the Free State is unacceptably low. Additional training should be implemented in the curricula and not only at an introductory level. Continuous training should be throughout the programme, which could also form part of the assessment in the final exam with a patient from the local communities. Burch has argued that medical curricula emphasise cultural competence but ‘have largely failed to address the challenge of non-English language competence of doctors’. She states that ‘the most basic need of a person seeking healthcare is the need to be heard and understood’.^21^ The use of interpreters would obviously assist with communication between the patient and doctor, but doctors being proficient in the language of their patients can only be advantageous. South Africa, with its 11 official languages (at the time of the study), is a challenging environment as students trained in a specific area, may receive training in languages common in that area (such as Afrikaans and Sesotho in the case of the Free State) but during their internship, community service and later career work in areas where other languages dominate. Adequate language proficiency training can help students become more culturally aware and adaptable to new circumstances.

### Limitations

Possible study limitations included that none of the authors are linguistic professionals. Technical difficulties could have influenced the telephonic interviews and the small sample of participants who consented to and participated in the interviews.

### Recommendations

It would be beneficial for UFS medical students in their clinical years to be able to communicate in Sesotho and Afrikaans. Incorporating training in these languages within the medical school curriculum is an option that the UFS may explore to address this issue. The level of language proficiency at the end of the language training and over the course and/or at the end of training is an avenue for future research.

Further research regarding the language proficiency of Sesotho and Afrikaans among different groups of medical students is also necessary to strengthen the need for an extensive review by the UFS regarding this matter. Alternatively, the UFS can require that prospective students have the basic language skills in Sesotho and Afrikaans before being accepted into the medical programme.

Students could be interviewed on what form of learning would be helpful for them to acquire the necessary language skills in Sesotho and Afrikaans.

We recommend that the telephonic interviews are recorded and analysed by professional linguistic practitioners in Sesotho and Afrikaans using more precise and practical tools for assessing language proficiency. Also, we recommend that the time schedules of the participants be considered when setting the times and dates for the telephonic interviews so that the researchers can know exactly when the participants are available for the telephonic interviews.

## Conclusion

The authors were able to assess Sesotho and Afrikaans language proficiency in a portion of fourth-year medical students; hence, the aim of the study was accomplished in this regard. Of the sample that underwent the entire course of this study (those whose language proficiency was tested via telephonic interviews), the majority were not proficient in Sesotho and Afrikaans. Only a single participant was assessed as proficient in both Sesotho and Afrikaans. This is a concern as Sesotho and Afrikaans are the most spoken languages in the Free State, making it challenging for medical students to communicate with the patients they encounter during their clinical rotations.

The current language training in Sesotho and Afrikaans in the undergraduate medical training at the UFS does not meet the objective of giving the students the language skills necessary to communicate with the majority of their patients in their home language.
